# Anxiety among Nursing Students towards Clinical Placement during COVID-19 in a Tertiary Hospital of Nepal: A Descriptive Cross-sectional Study

**DOI:** 10.31729/jnma.6132

**Published:** 2021-06-30

**Authors:** Bidhya Banstola, Nona Shakya, Pushpa Sharma

**Affiliations:** 1 Nursing Programme, Manipal College of Medicai Sciences, Pokhara, Nepal

**Keywords:** *anxiety*, *coping*, *COVID-19*, *nursing students*

## Abstract

**Introduction:**

The world is facing the pandemic of COVID-19 caused by the corona virus since December 2019 and has caused millions of death throughout the world. Exposure of nursing students in clinical placement during pandemic is fearful and stressful with high risk of infection which can cause anxiety and different levels of psychological crisis to individuals. The main objective of the study is to find out the prevalence of anxiety among nursing students during clinical placement in the pandemic of COVID-19.

**Methods:**

A descriptive cross-sectional study was conducted among 144 nursing students enrolled in different clinical placement of a tertiary hospital of Nepal from 20th January 2021 to 2nd February 2021. Ethical approval was received from the Institutional Review Committee. Demographic, COVID-19 related and Beck Anxiety Inventory questionnaires was used for assessing anxiety. Whole sampling was done. Descriptive statistics was conducted using Statistical Package for the Social Sciences 2016 version.

**Results:**

Out of 144 females enrolled in the study, all the nursing students 144 (100%) having clinical placement had anxiety. Among them, 117 (81%) had mild anxiety and 27 (19%) had moderate level of anxiety. All the students used coping strategies for the anxiety. The most commonly used strategy to cope with anxiety was religion (5.03±1.78).

**Conclusions:**

All the nursing students had anxiety during the clinical placement and all the students used the coping strategies for the anxiety. Majority of the nursing students had mild anxiety. Religion was most common method of strategy to cope with anxiety.

## INTRODUCTION

The world has been facing the COVID-19 pandemic since December 2019. Nursing students are in close contact with the patients in providing continuous care during their clinical placement. Literatures have shown that practical training has become more stressful than academic training.^1^

Anxiety is an unpleasant emotional state or reaction with the subjective feeling of tension, apprehension, discomfort, worry and nervousness which is different to individual response usually caused by the anticipation of fear.^2^ The coping strategies that are used by the nursing students during the care of COVID-19 patients have been found to be immature and negative whereas the nurses have used positive coping mechanism.^3^ Studies on anxiety and coping strategies have been few in Nepal.

This study aimed to find out the prevalence of anxiety among nursing students towards clinical placement in the pandemic of COVID-19 in a tertiary care hospital.

## METHODS

A descriptive cross-sectional study was conducted from 20th January 2021 to 2nd February 2021 among 144 nursing students working in different departments of Manipal College of Medical Sciences in Pokhara. The ethical clearance for conducting the study was taken from the Institutional Review Committee of Manipal Teaching Hospital (Ref: MEMG/IRC/431/GA). Nursing students who were having clinical placement during the time of data collection and who had no known mental illness were included in the study. Forty nursing students from PCL 2nd year were excluded from the study since they had already completed their clinical placement during the time of data collection.

The whole sampling was done among the nursing students having clinical placement in a tertiary care hospital. Out of 192 nursing students from Manipal college of Medical Sciences 152 were eligible for the study since they were having their clinical placement during the time of data collection. The eligible students were sent the google link to the online questionnaire. Out of 152 eligible nursing students, only 144 filled and sent the questionnaire back giving the response rate of 95%.

The self-administered online questionnaire consisted of demographic, clinical and COVID-19 related questions with 10 items; a standard tool as a Beck Anxiety Inventory^4^ having 21 items to assess the symptoms of anxiety and Brief-COPE^5^ with 28 items to assess the coping strategies. Anxiety was assessed based on 4 points Likert scale assigned as the score of 0,1,2,3 respectively for not at all, mildly, but it didn't bother me much, Moderately, it wasn't pleasant at times and severely-it bothered me a lot. Those who scored 0-21 were considered as mild or low anxiety; 22-35 as moderate anxiety and those who scored 36 and above as severe anxiety. Likewise, the coping strategies with 4 points Likert scale was assigned a score of 1,2,3 and 4 respectively for I haven't been doing this at all, a little bit medium amount and I have been doing this a lot. The various coping strategies were broadly categorized into approach coping, avoidant coping, humour and religion. Various items in the tool were clubbed as avoidant coping which were identified as denial, substance use, venting, behavioural disengagement, self-distraction and selfblame. Active coping, positive reframing, planning, acceptance, seeking emotional support and seeking informational support were the strategies that were clubbed for various items and identified as approach coping.

Informed consent was taken from the respondents via an online google link before data collection. Reading the informed consent and clicking the agree option denoted their consent to get involved in the study.

The respondents were sent an online questionnaire via messenger in a group and the link was resent after a week to remind them. The data retrieved from the google form were transferred to excel and then to Statistical Package for the Social Sciences (SPSS) software 16 for analysis. The data were analyzed using descriptive statistics like frequency, percentage, mean and standard deviation.

## RESULTS

Out of 144 females enrolled in the study, all the nursing students 144 (100%) having clinical placement in the pandemic of COVID-19 had anxiety. All 144 (100%) nursing students coping strategies for anxiety during clinical placement. Among them, 117 (81%) had mild anxiety and 27 (19%) had a moderate level of anxiety. The mean age of the nursing students participating in this study was 19.55±1.7 years with ages ranging from 16-22 years. Almost 3/4th of the respondents were from inside the district. Around 77 (53.5%) of the respondents were from proficiency certificate level (PCL). The majority of the respondents 40 (27.8%) were from the academic year of PCL 1st year (Table 1).

**Table 1:** Demographic characteristics of the respondents (n= 144).

Demographic Characteristics		n (%)
Place of Residence	Inside the district	107 (74.3)
	Outside the district	37 (25.7)
Educational Program	Proficiency Certificate Level	77 (53.5)
	BSc Nursing	67 (46.5)
Academic Year	PCL 1st Year	40 (27.8)
	PCL 3rd Year	37 (25.6)
	BSc 1st Year	15 (10.4)
	BSc 2nd Year	26 (18.1)
	BSc 3rd Year	26 (18.1)

Three (2.1%) of the students had a personal history of COVID-19 infections. The majority of the students 127 (88.2%) stayed in quarantine before starting the clinical placement and almost 128 (88.9%) of the respondents were knowledgeable on the use of personal protective equipment. Almost all 133 (92.4%) respondents had fear related to the outcome of clinical placement (Table 2).

**Table 2:** Clinical placement and COVID-19 related characteristics of the respondents (n= 144).

Characteristics	n (%)
Personal History of COVID-19 Infections	3(2.1)
Family History of COVID-19 Infections	15 (10.4)
Quarantine Status before Clinical Placement	127 (88.2)
Knowledge on use of Personal Protective Equipment	128 (88.9)
Knowledge on the current status of COVID-19	137 (95.1)
Fear related to the outcome of clinical	133 (92.4)

Severe symptoms of anxiety were not present commonly. The majority of the symptoms were mildly present. Some symptoms like nervousness were reported to be moderately present in 36 (25%) respondents (Table 3).

**Table 3:** Anxiety symptoms of the respondents (n= 144).

Symptoms	Not at all n (%)	Mildly n (%)	Moderately n (%)	Severely n (%)
Numbness or tingling	91 (63.2)	46 (31.9)	7 (4.9)	
Feeling hot	89 (61.8)	40 (27.8)	14 (9.7)	1 (0.7)
Unable to relax	64 (44.4)	43 (29.9)	29 (20.1)	8 (5.6)
Fear of worst happening	33 (22.9)	75 (52.1)	24 (16.7)	12 (8.3)
Dizzy or light headed	46 (31.9)	77 (53.5)	16 (11.1)	5 (3.5)
Heart pounding/racing	77 (53.5)	44 (30.6)	20 (13.9)	3 (2.1)
Unsteady	103 (71.5)	31 (21.5)	9 (6.3)	1 (0.7)
Terrified/afraid	46 (31.9)	51 (35.4)	36 (25.0)	11 (7.6)
Nervous	39 (27.1)	61 (42.4)	35 (24.3)	9 (6.3)
Feeling of choking	119 (82.6)	21(14.6)	4 (2.8)	
Hands trembling	106 (73.6)	34 (23.6)	3 (2.1)	1 (0.7)
Shaky/unsteady	107 (74.3)	32 (22.2)	4 (2.8)	1 (0.7)
Fear of losing control	91 (63.2)	36 (25.0)	13 (9.0)	4 (2.8)
Difficulty in breathing	96 (66.7)	27 (18.8)	20 (13.9)	1 (0.7)
Fear of dying	82 (56.9)	39 (27.1)	13 (9.0)	10 (6.9)
Scared	40 (27.8)	75 (52.1)	14 (9.7)	15 (10.4)
Indigestion	78 (54.2)	42 (29.2)	18 (12.5)	6 (4.2)
Faint/Lightheaded	97 (67.4)	36 (25.0)	9 (6.3)	2 (1.4)
Face flushed	100 (69.4)	25 (17.4)	17 (11.8)	2 (1.4)
Hot/cold sweats	84 (58.3)	45(31.3)	14 (9.7)	1 (0.7)

**Figure 1:**
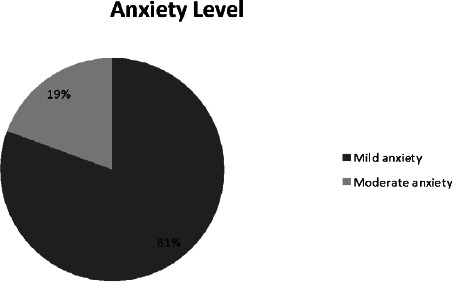
Level of anxiety of respondents

The majority of the respondents 117 (81%) had a mild or low level of anxiety (Figure 1).

The most commonly used strategy to cope was religion and humour was the least used. Acceptances, seeking informational and emotional support were the highly used approach coping strategies. The respondents used denial and self-distraction most often as avoidant coping strategies to cope with anxiety (Table 4).

**Table 4:** Coping strategies adopted by the respondents (n = 144).

Coping Strategies		Mean±SD
Religion		5.03±1.78
Approach Coping	Acceptance	2.78±0.89
	Seeking Informational Support	2.66±0.92
	Seeking Emotional Support	2.64±0.82
	Positive Reframing	2.62±0.90
	Active Coping	2.55±0.80
	Planning	2.43±0.83
Avoidant Coping	Denial	3.33±1.56
	Self-Distraction	2.25±0.77
	Venting	1.95±0.70
	Behavioural disengagement	1.74±0.80
	Self-Blame	1.66±0.67
	Substance Use	1.10±0.30
Humor		1.28±0.50

## DISCUSSION

In nursing, the topic of stress has got more attention which is evident in various literatures.^6,7^ Exposure of nursing students to an epidemic or pandemic is stressful for them with the fear of getting an infection.^8^ The reluctance to work in health care facilities by health care providers and nursing students with the fear of the high risk of infection with inadequate infection control and isolation measures is evident from the study done in Saudi Arabia during the MERS outbreak in 2016,^8^ SARS outbreak in 2003 in Hongkong.^9^ COVID-19 is a source of stress to an individual and group of people and they may have different levels of psychological crisis differently and those nurses who are at the core of the incident are more affected.^3^

The prevalence of low and moderate anxiety during clinical placement in COVID-19 among nursing students was found to be 19% and 81% respectively in this study. This finding varies from the study conducted in Ashkelon Academic College, Southern District, Israel which showed the prevalence of moderate and severe anxiety was 43% and 13% respectively.^10^ Such a variation in the level of anxiety may be due to the timing of the study conducted. The current study was conducted after nine months of the emergence of the pandemic however the study conducted in Israel was during the third week of lockdown. Likewise, the study conducted in College of Medical Applied Sciences Mohali Asser-King Khalid University showed that there is a prevalence of mild and moderate anxiety which is consistent with the findings of this study.^11^ The findings of this study is also inconsistent with the study done in Assiut University and Indonesia which showed that nursing students also have severe anxiety along with the mild and moderate level of anxiety.^12,13^

The most common symptoms of moderate anxiety were terrified/afraid, nervous and unable to relax which were present among 25%, 24.3% and 20.1% respectively. This finding is consistent with the study done in Assiut University which showed that 21.3% of nursing students had fear and 36.1% were anxious.^12^

In this study, the most commonly used strategies to cope were religion (5.03%), denial (3.33%) and acceptance (2.78%). Religion was also the most frequent coping strategy used by undergraduate Malaysian students during their clinical practice.^14^ However the study conducted in Israel revealed that coping strategies like humour and mental disengagement coping strategies were used more to overcome the anxiety.^10^ This variation may be due to the difference in the anxiety level as a study in Israel was conducted during the early phase of lockdown. The least commonly adapted coping styles of the participants were substance use (1.1%), humour (1.28%) and self-blame (1.66%). These findings are in contrast with the coping styles among Japanese Nursing Students in which the three most common coping styles adopted by the participants were acceptance (5.7%), self-distraction (5.5%) and planning (5.4%). The least common coping styles were substance use, religion, and denial.^15^ The difference in the score and coping strategies are likely due to the difference in the settings in which the studies were conducted and also the difference in culture may have made an impact too.

Except for the denial coping strategy, this study showed that nursing students used adaptive coping skills more often than avoiding coping skills. Students were involved more in positive coping methods like active coping, positive reframing, seeking emotional support and planning in COVID times. This finding is similar to a study done among undergraduate nursing students in Western Rajasthan.^16^ As the lockdown and the emergence of cases were similar in India and Nepal with similar culture and methods of teaching too, the results were found to be consistent. These findings are also in consistent with other study done in Jimma University in Ethiopia which reported the use of healthy coping strategies as the common method of coping.^17^

The self-reported data may lead to the over or underestimation of the anxiety and coping strategies. Since this is a single-centre study with a medium sample size also doesn't allow for generalisation of the findings of the study in the whole population.

## CONCLUSIONS

Anxiety and coping strategies during the clinical placement was present in all the nursing students during COVID-19 which is high. The anxiety of nursing students during the clinical placement in the pandemic of COVID-19 was found at a mild level in most of the students. The most strategies that were used to cope with anxiety were religion, acceptance and denial. Anxiety can affect the health of nursing students so identifying the needs of the students in the clinical setting and developing interventions to reduce the possible anxiety and adopt adaptive coping strategies will be helpful to reduce mental health problems associated with the pandemic.
